# Uncovering the structural impact of KatG Ser315 mutations in *Mycobacterium tuberculosis* via cryo‐EM

**DOI:** 10.1002/pro.70409

**Published:** 2025-12-23

**Authors:** Thomas Allport, Amanda K. Chaplin

**Affiliations:** ^1^ Department of Molecular and Cell Biology, University of Leicester Leicester Institute for Structural and Chemical Biology Leicester UK

**Keywords:** catalase, cryo‐EM, heme, KatG, peroxidase

## Abstract

*Mycobacterium tuberculosis* (*Mtb*), the causative agent of tuberculosis (TB), is responsible for a global health burden affecting over a quarter of the world's population. The increasing prevalence of drug‐resistant TB poses a significant threat to current treatment strategies. Isoniazid (INH) is a first‐line prodrug used in TB therapy, which requires activation by the catalase‐peroxidase enzyme KatG. Upon activation, INH inhibits InhA, thereby disrupting mycolic acid biosynthesis, a crucial process for maintaining *Mtb*'s distinctive, lipid‐rich cell wall. The most common naturally occurring resistance‐associated mutation in KatG is S315T, though other variants at this position, such as S315G, S315N, S315I, and S315R, have also been reported. In this study, we employ cryo‐electron microscopy (cryo‐EM) to investigate the structural basis of INH resistance conferred by these KatG variants. We present high‐resolution cryo‐EM structures that reveal heterogeneity in heme loading among the mutants. Detailed structural analysis highlights alterations in the hydrogen‐bonding network and substrate access channel unique to each variant, offering direct comparisons with the wild‐type (WT) KatG protein. Our findings provide a molecular explanation for clinical INH resistance and lay the groundwork for the rational design of next‐generation anti‐TB therapeutics.

## 1 INTRODUCTION

*Mycobacterium tuberculosis* (*Mtb*) is the causative agent of the respiratory disease tuberculosis (TB), which is estimated to have infected one quarter of the global population (Bagcchi, 2023). The bacterium also has the capability to persist in the infected human for decades. There is a serious public health crisis surrounding TB, which is intensified largely by the increasing number of cases of multi‐drug‐resistant TB (MDR‐TB).

The *Mtb* protein KatG is a 160 kDa bifunctional catalase‐peroxidase heme enzyme, which is critical for the activation of isoniazid (INH), one of the first‐line pro‐drugs utilized for treatment of TB. INH has been used as treatment for TB for over 60 years, since its FDA approval in 1952 (Hameed et al., 2020; Jagielski et al., 2014; Kidenya et al., 2018; Torres, Garcia‐Garcia, et al., 2015; Torres, Paul, et al., 2015). The ability of KatG to utilize hydrogen peroxide (H_2_O_2_) either through functioning as a catalase or as a peroxidase has been studied for several decades (Dunford, 2010). The peroxidase activity of the enzyme is essential for activating INH, whereas its catalase activity protects *Mtb* from oxidative stress (Figure S1). Given the importance of KatG for INH activation, it is perhaps unsurprising that over half of all known INH resistance mutations are found within the *katG* gene (Cardoso et al., 2004; Hazbon et al., 2006; Muthaiah et al., 2017; Zhang et al., 2005). However, in general, the study of resistance mutations in KatG has shown that there is no correlation between catalase or peroxidase activities and INH resistance in KatG (Cade et al., 2010). Once KatG activates INH, the drug forms an adduct with nicotinamide adenine dinucleotide (NAD) to form the isonicotinoyl‐NAD adduct (INH‐NAD) that targets InhA (2‐trans‐enoyl‐acyl carrier protein reductase) (Figure S1). Inhibition of InhA disrupts mycolic acid (MA) biosynthesis, a key component of the characteristic thick cell wall of the bacterium which promotes cellular survival in the adverse conditions encountered during human infection. For the development of future anti‐TB therapies, it is therefore paramount we understand how these resistance mutations in KatG affect the ability of the enzyme to activate INH. Although there are drugs in addition to INH which are used to treat TB, many treatments last months, cause severe side effects and lead to MDR‐TB. Resistance to INH and/or other anti‐TB drugs has a critical impact on the ability to treat and eradicate this disease, and further investigations are required to combat the effect of these mutations.

The most common resistance mutation identified in KatG is the substitution of Ser 315 to Thr and is shown to significantly impair INH activation (Kapetanaki et al., 2003; Kapetanaki et al., 2005; Mokrousov et al., 2002). S315T disrupts the substrate access channel, reducing INH activation by approximately 5‐fold (Cade et al., 2010; Saint‐Joanis et al., 1999). S315T is commonly observed in MDR and extensively drug‐resistant (XDR) strains. INH resistance has also been observed in strains with alternative amino acid substitutions of S315, including S315N, S315R, S315I, and S315G (Bakhtiyariniya et al., 2022; Cardoso et al., 2004; Pinhata et al., 2021). Recently, an in silico docking study looked into these mutations and simulated with 50 INH derivatives in comparison to wild‐type (WT) KatG (Unissa et al., 2017). It was found that a derivative of INH, C50 [isonicotinic acid (2‐hydroxy‐3‐methoxybenzylidene) hydrazide], can be considered a better lead for INH‐resistant strains. However, advancement in compounds and future drug treatment requires experimental and structural data on these KatG resistance mutations (Unissa et al., 2017).

Recently, our group utilized the advancements in cryo electron microscopy (cryo‐EM) to study resistance mutations and INH binding in KatG (Munir et al., 2021). In this study, we solved a high‐resolution cryo‐EM structure of KatG in the presence of INH, which together with docking studies identified a potential binding pocket for INH. The proposed INH binding site (denoted site 1) is located at the entrance to the heme pocket, in the region of the aforementioned residue Ser 315, and additionally Asp 137, with both of these residues previously reported to regulate INH activation (Zhao et al., 2013). We also investigated two INH resistance mutations (T275P and W107R), observing their ability to induce structural disorder and reduction of heme binding and retention, necessary for enzyme activity and INH activation (Munir et al., 2021).

In this study, we utilize our optimized cryo‐EM methodology for obtaining KatG structures and present high‐resolution structures of the variants S315T, S315I, S315N, S315R, and S315G. We discuss the variable heme loading (how much heme is able to load) in these mutant proteins and discuss the disruption of the major INH binding site and substrate access channel. We finally conclude the effect these variants have on INH activation and discuss how this experimental information could lead to future therapeutics. Understanding drug resistance in KatG will allow for the potential use of existing or new anti‐TB drugs that are active against INH‐resistant TB strains.

## 2 RESULTS

**FIGURE 1:**
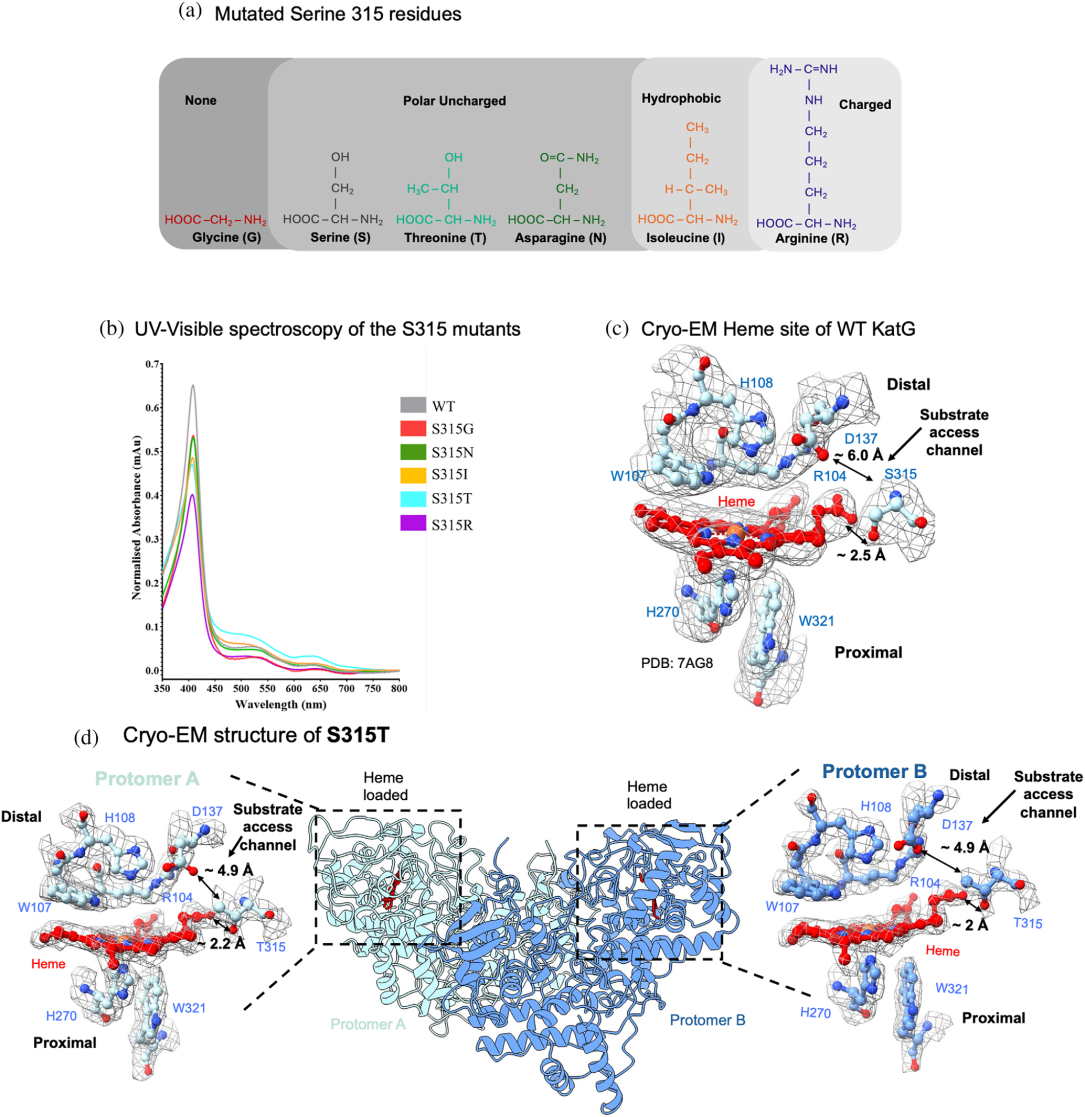
KatG Ser315 variants. (a) KatG S315 variant side chains, with amino acid side chains shown and colored according to B. (b) UV–visible spectroscopy of WT KatG and S315 variants. (c) Close up heme map and structure of WT KatG (PDB: 7AG8) (Munir et al., 2021). Heme is shown in red and side chains blue and labeled. (d) Cryo‐EM structure of S315T to 2.2 Å resolution. Protomer A is shown in light blue and protomer B in dark blue. Close up of the heme sites are also shown with heme in red and residues in blue and labeled.

The five naturally occurring resistance mutations at the S315 position represent a range of amino acids with differing chemical properties (Figure 1a). Glycine (G) is the simplest mutation, with no side chain present. Whereas Serine (S) (present in the WT protein), Threonine (T), and Asparagine (N) display polar uncharged side chains. Isoleucine (I) consists of a slightly larger hydrophobic side chain, and Arginine (R) contains a large electrically charged side chain (Figure 1a). Following successful purification of the WT KatG protein and the mutant proteins, high purity was confirmed through SDS‐PAGE gel analysis (Figure S2).

### 2.1 UV–visible spectroscopy of the KatG variants shows variable heme loading

To first characterize these S315 variants, we measured the UV–visible absorbance spectrum of all five variants and compared these to the WT KatG protein (Figure 1b). All proteins were brown in color and gave rise to transitions in the electronic absorbance spectrum. These electronic transitions arise from the bound b‐type heme and generate wavelengths consistent with a ferric (Fe^3+^) resting state catalase‐peroxidase enzyme. These spectral transitions visualized in the visible region comprise a Soret peak (arising from π–π* transitions), common in proteins containing a coordinated heme, between ~406–409 nm and *Q* charge transfer bands with specific wavelengths recorded in Table S1 (Johnsson et al., 1997). The variant proteins gave rise to subtle changes in the Soret and charge transfer bands indicative of the change in the electronic environment surrounding the heme site.

Purified WT KatG has a Reinheitszahl ratio (*R*_Z_ = *A*_407_/*A*_280_) of 0.652, which is within the range of previously reported values for fully loaded heme WT protein (Ghiladi et al., 2005; Munir et al., 2021; Wengenack et al., 2000). Interestingly, all the KatG mutants displayed a lower *R*_z_ than the WT protein. Ranging from 0.40 in S315R to 0.54 in S315G (Table 1). It should be noted that these variants were exposed to a large excess of heme during purification, identical to that of the WT protein. It should also be mentioned that these variant proteins were desalted, to remove any excess heme (see methods), and the *R*_z_ values also appear lower than the WT. Although the *R*_z_ values (and therefore heme loading) for the variant proteins varied slightly depending on the purification, they were always consistently lower than the WT protein. This therefore shows that mutations at S315 within KatG cause heme loading and retention (heme being loaded and also retained and not removed) to be lower than that of the WT protein, a feature that we have reported and discussed for other KatG variants.

**TABLE 1:** Comparative summary of the characteristics of S315 WT and variants.

Category						
Variant	Gly (G)	**Ser (S)**	Ile (I)	Thr (T)	Asn (N)	Arg (R)
Rz	0.536	**0.652**	0.485	0.468	0.531	0.4
Substrate access channel (Å)	~7	**~6**	~4.2	~4.9	~3	~3
Caver average radius (Å)	1.8	**1.7**	1.03	1.38	1.15	0.9
Cryo‐EM maps (0:1:2 heme)	1 1 0	**0 0 1**	1 1 1	0 0 1	1 1 0	0 1 1
INH adduct formation (120 μM 1 h) (%)	102.8	**100**	106.7	23.8	14.5	6.5

*Note*: WT is colored in green text and bold. They are grouped by similarity highlighted with either blue or green. INH adduct formation has come from Cade et al. (2010).

### 2.2 S315T constricts the substrate access channel

The most common KatG resistance mutation is S315T found in a range of patients 52%–84.3% (Alagappan et al., 2018; Asante‐Poku et al., 2015; Bakhtiyariniya et al., 2022; Nono et al., 2025). Previously, the x‐ray crystal structure of the S315T has been solved to 2.1 Å resolution (Zhao et al., 2006). However, as we found with our previous KatG studies using cryo‐EM, the ability to capture heterogeneity in samples that can be associated with conformational changes undetected by x‐ray crystallography holds a great advantage, and thus we have continued the cryo‐EM approach here. The S315T variant for cryo‐EM studies was prepared as previously described with the addition of 3‐[(3‐cholamidopropyl) dimethylammonio]‐1‐propanesulfonate (CHAPSO) to prevent particle orientation bias (Munir et al., 2021). Cryo‐EM micrographs showed clear particle distribution and contrast (Figure S4). Following data collection and processing, we resolved a map to 2.2 Å resolution (Table S2, Figures 1d and S3, S5, S6). In this cryo‐EM map, we were able to model heme into both protomers of the homodimer. This, therefore, shows that the S315T variant is clearly loaded with heme, like the WT protein showed previously (Munir et al., 2021).

The active site residues surrounding the heme remain identical to WT and the previous x‐ray crystallography structure of S315T (Figures 1c,d, S3, S4, S5). The heme is pentacoordinate as shown in Figure 1d, with His 270 coordinating the heme Fe on the proximal side. In addition, the heme site is surrounded by five other conserved residues: Trp 321 and Asp 381 in the proximal site and Arg 104, Trp 107, and His 108 in the distal pocket. On the distal side of the heme, density can be seen for the formation of the previously observed MYW catalytic triad of Met 255, Tyr 229, and Trp107. This is a post‐translational modification where these three amino acids become crosslinked.

The distal side of the heme pocket is connected to the surface through a long substrate access channel lined with Thr315 and Asp137, creating the narrowest point close to the edge of the heme. This narrow point in the substrate (INH) access channel creates a steric barrier for access to the heme site. The most significant change compared to the WT protein is the presence of the methyl group of the Thr315 side chain. The methyl group present in the S315T variant effectively constricts the substrate access channel to the heme, with a distance between Asp137 and Thr315 ~4.9 Å, similar to that previously measured for the x‐ray crystal structure of S315T (~4.7 Å) (Zhao et al., 2006). This compares to ~6 Å for this distance between Ser315 and Asp137 in the WT protein (Figure 1c) (Munir et al., 2021; Zhao et al., 2006). CAVER analysis predicts an average radius of 1.38 Å in S315T compared to 1.7 Å in the WT protein, again confirming the restriction of the channel from the replacement of a Ser to Thr residue (Table 1) (Chovancova et al., 2012). Interestingly, S315T still retains the hydrogen bond between Thr315 and the heme propionate with a distance of ~2 Å, like that seen in the WT protein (Figures 1c,d, S7). Therefore, although the cryo‐EM structure of S315T is similar to that of the x‐ray crystallography structure, it confirms our ability to use cryo‐EM to study single point mutations in KatG, allowing us to study the other additional S315 mutants.

### 2.3 Cryo‐EM of Ser315 KatG variants show variable heme loading

**FIGURE 2:**
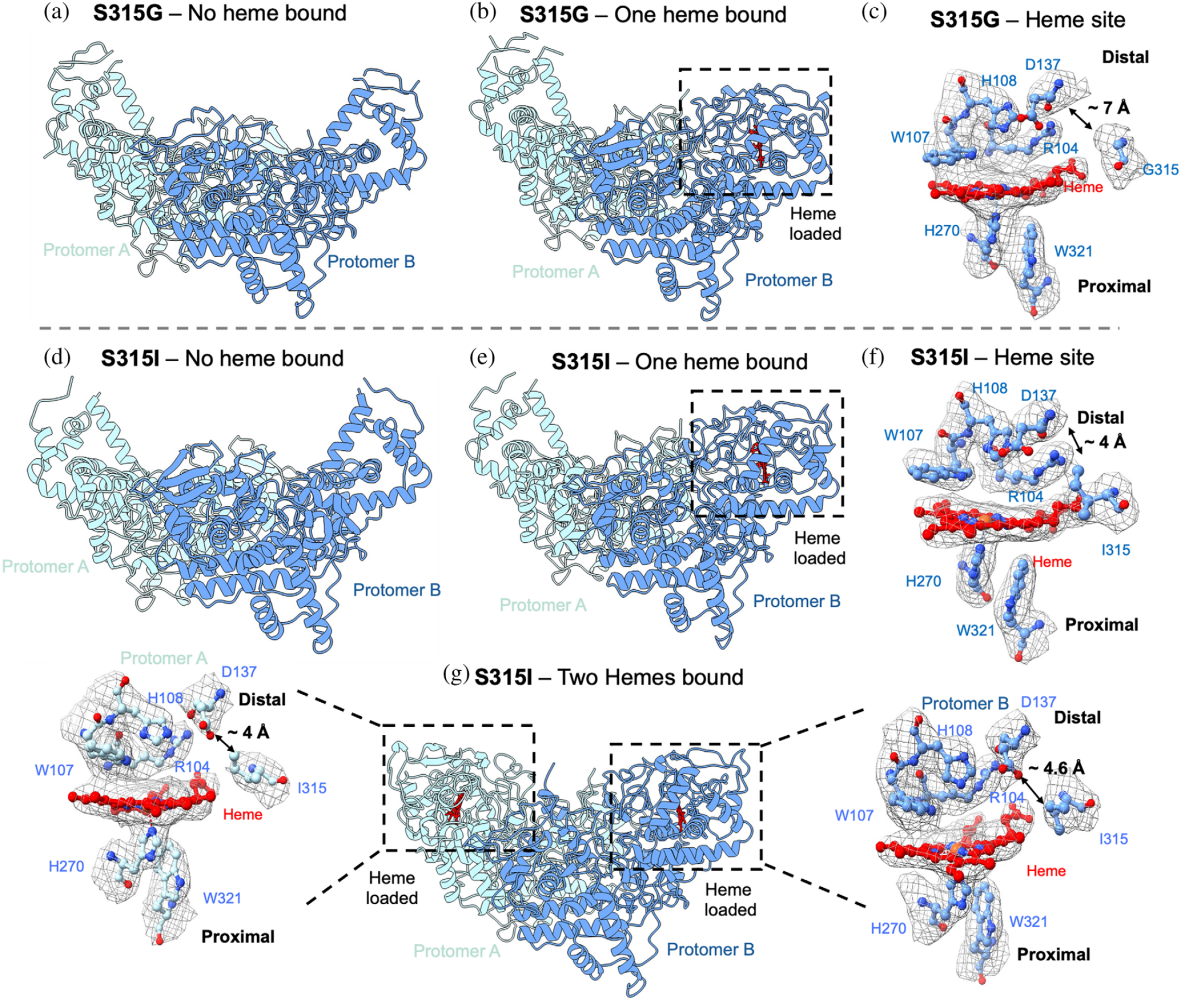
Cryo‐EM structures of S315G and S315I KatG variants. (a), (b) Show the overall cryo‐EM map and modeled structure for S315G no heme bound to 2.7 Å resolution and one heme bound to 2.7 Å resolution, respectively. Protomer A is shown in light blue and protomer B in dark blue. (c) Close up of the heme site for S315G, residues and important distances labeled. (d), (e), (g) Show the overall cryo‐EM map and modeled structure for S315I no heme bound to 3.0 Å resolution, one heme bound to 2.3 Å resolution and two hemes bound to 2.8 Å resolution, respectively. Protomer A is shown in light blue and protomer B in dark blue. (f and insets) Close up of the heme site for S315I, residues and important distances labeled.

**FIGURE 3:**
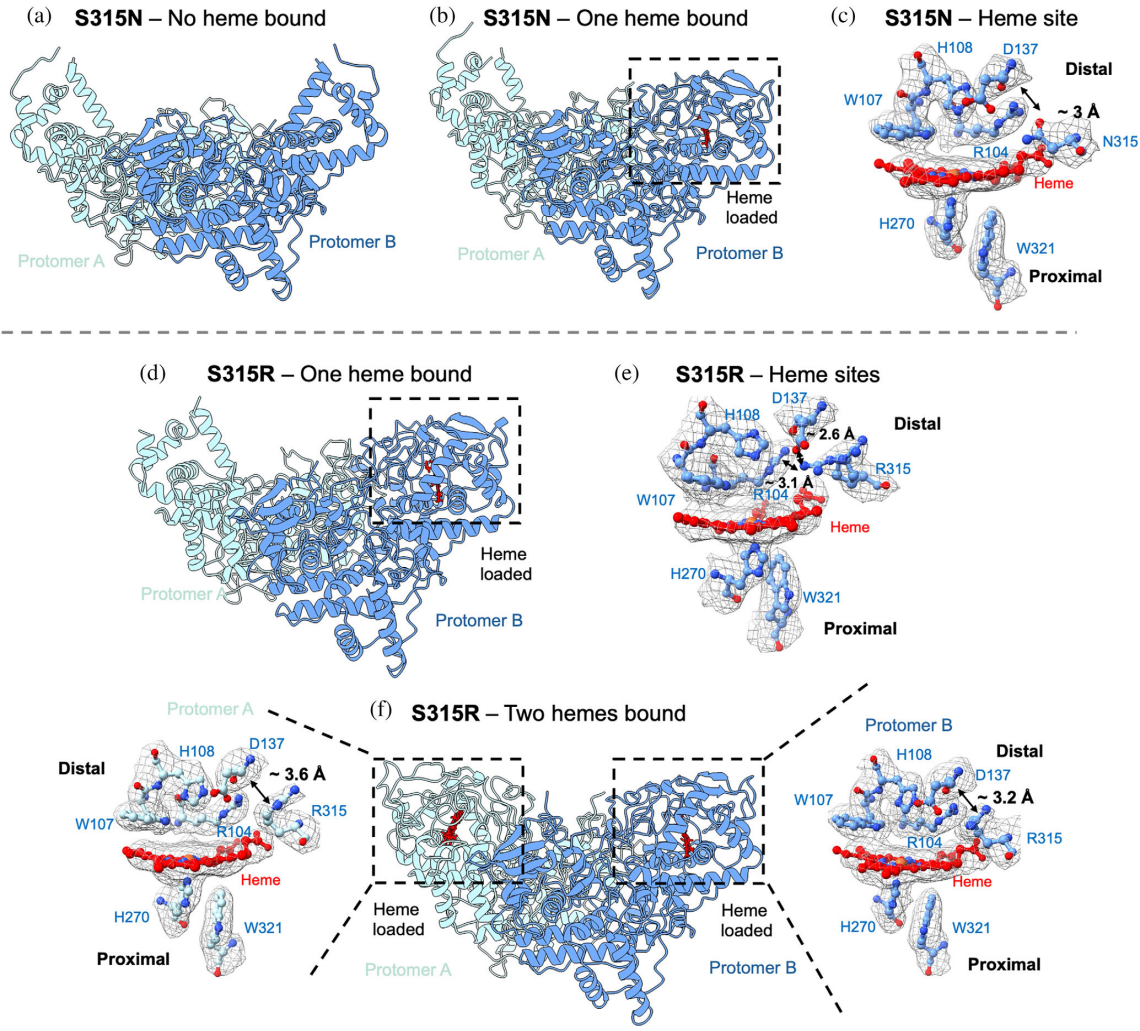
Cryo‐EM structures of S315N and S315R KatG variants. (a), (b) Show the overall cryo‐EM map and modeled structure for S315N no heme bound to 2.6 Å resolution and one heme bound to 2.6 Å resolution, respectively. Protomer A is shown in light blue and protomer B in dark blue. (c) Close up of the heme site for S315N, residues and important distances labeled. (d), (f) Show the overall cryo‐EM map and modeled structure for S315R with one heme bound to 2.7 Å resolution and two hemes bound to 2.6 Å resolution, respectively. Protomer A is shown in light blue and protomer B in dark blue. (e) and (f, insets) Close up of the heme site for S315R, residues and important distances labeled.

Following analysis of S315T, we wanted to structurally analyze using cryo‐EM the other four KatG variants, S315G, S315N, S315I, and S315R. These variants were prepared in an identical manner to the WT protein, with excess heme loading during purification. All variants again produced good particle distribution and contrast (Figure S4), highlighting the capability of using cryo‐EM for analysis of a variety of KatG mutants. Following data collection and processing, it was clear that the cryo‐EM samples were heterogeneous, producing a mixture of cryo‐EM maps. Some data produced maps where heme is present in both protomers of the homodimer, some where it is only present in one protomer, and others where heme was not present in either protomer (Figures 2, 3). A summary of these ratios and particle numbers is given in Tables 1, S2. This was different from that previously observed for the WT and S315T KatG proteins, where the majority of particles were present with heme in both protomers (Munir et al., 2021).

Based on prior enzymatic assays (peroxidase and catalase) and INH‐adduct formation data from a previous study Tables 1 (blue, green), S4, S5, the S315G and S315I mutants exhibited activity profiles more closely resembling the WT protein, in contrast to the S315N, S315R, and S315T variants (Cade et al., 2010). We, therefore, first explored cryo‐EM structures of S315G and S315I.

### 2.4 S315G displays a larger substrate access channel

S315G contains the simplest mutation, replacing the serine residue with a glycine (Gly, G). In this dataset, two maps were resolved: one with one heme loaded to 2.7 Å resolution and the other with no heme loaded to 2.7 Å resolution (Figure 2a,b and S3, S8). This lack of heme loading is perhaps surprising, given that this variant produced an *R*_z_ value closest to the WT protein (0.536 in S315G compared to 0.652 WT (Table 1)). With the lack of a side chain at this position, the tunnel/pocket leading to the heme site is no longer constricted to such a degree as shown in the WT protein and the S315T variant. With the narrowest part of the channel measuring as 6 Å in WT and 4.9 Å in S315T, compared to ~7 Å in the S315G one heme model. This was also calculated using CAVER analysis, showing the increase in size of ~1.8 Å (Figures 2c, S9, Table 1) (Chovancova et al., 2012). There is also no longer a hydrogen bond between S315 and the heme propionate. It is plausible that this large pocket allows for heme loading (shown through the UV–visible spectrum) but lacks retention (identified through the cryo‐EM structures).

### 2.5 S315I loses hydrogen bonding and restricts the substrate access channel

We then replaced Ser315 with isoleucine (Ile, I), a large but hydrophobic amino acid side chain. Following data collection and processing, we were able to resolve three maps, one with heme loaded in both protomers to 2.8 Å resolution, one with one heme loaded to 2.3 Å resolution, and the other with no heme loaded to 3.0 Å resolution (Figure 2d,e,g and S3, S10). Due to the lack of polarity of this side chain, like S315G, the hydrogen bonding has been lost, with a distance between the isoleucine side chain and Asp137 of ~4.2 Å. This compares to ~7 Å in S315G, ~6 Å in WT, and is more like S315T ~4.9 Å. The hydrogen bond to the heme has also been lost due to the hydrophobic side chain. This therefore shows that, although there is no H‐bonding, the substrate access channel has been constricted. This was also confirmed again through CAVER analysis with a radius of 1.03 Å (Table 1) (Chovancova et al., 2012).

### 2.6 S315N constricts the substrate access channel and hydrogen bonds to Asp137

We next wanted to explore the S315 variants, which show low INH adduct formation similar to S315T, which include S315N and S315R (Figure 3, Table 1).

We therefore first replaced Ser315 with an Asn residue which contains a carboxamide (or amide) side chain, −(CH_2_)–CO–NH_2_, which is polar and can participate in hydrogen bond formation. Following cryo‐EM data collection and processing, we were able to resolve two maps, one with one heme loaded to 2.6 Å resolution and the other with no heme loaded to 2.6 Å resolution (Figures 3a,b and S3, S11). This variant had an *R*_z_ value of 0.531, similar to S315G (0.536), which also produces a no heme and a one heme map. Not surprisingly, CAVER analysis showed that this variant has a more constricted substrate access channel compared to WT, of 1.15 Å compared to 1.7 Å in WT.

Upon comparing the one heme loaded structure with that of the WT, the Asn side chain can be seen close to the Asp137, with a distance between the NH_2_ group of N315 and the oxygen side chain of Asp137 of ~3 Å, indicative of a potential hydrogen bond (Figure 3c). This distance differs greatly in the S315G mutant, which is ~7 Å; however, similar heme loading is identified with the cryo‐EM maps, highlighting that the size of this amino acid does not dictate heme loading. No hydrogen bonding is observed with the heme.

### 2.7 S315R restricts the substrate access channel and retains heme

Finally, we replaced Ser315 with an Arg, R, which contains a large, charged side chain. Following data collection and processing, we were able to resolve two maps: one with heme loaded in both protomers to 2.6 Å resolution, one with one heme loaded to 2.7 Å resolution (Figures 3d,f and S3, S12). This observed increase in heme loading is contradictory compared to the UV–visible spectrum of this variant, which displayed the lowest *R*_z_ value of all the variant proteins (0.4, Table 1). It also highlights, together with data from S315I, that the larger the amino acid side, the more restricted heme is and the more likely it is retained within the protein.

In the one heme bound structure, the Arg side chain could be modeled in two orientations, one toward the heme site and the second protruding into the substrate access channel (Figure 3e). The Arg pointing toward the heme forms several H‐bonds with Asp 137 and Arg 104, whereas the second conformer does not form any. In the two heme bound maps, the Arg side chain has only one orientation predominantly pointed away from the heme site with a distance to Asp 137 of ~3.6 Å in protomer A and ~3.2 Å in protomer B (Figure 3f). The average CAVER radius is 0.9 Å, the lowest of all the KatG variants, due to the increased bulk of this side chain (Table 1) (Chovancova et al., 2012).

## 3 DISCUSSION

### 3.1 Complexity of INH resistance conferred by S315 mutations

Our findings highlight the complexity of INH resistance associated with mutations at residue S315 in KatG. Any substitution at this position appears to confer resistance, yet the underlying mechanisms vary depending on the specific mutation. In the cases of S315G and S315I, the mutations either expand or constrict the substrate access channel, respectively, both resulting in disruption of the H‐bonding network with the heme and ultimately eliminating any H‐bonding interactions. In contrast, S315N and S315R not only constrict the substrate channel but also alter the H‐bonding pattern, which likely exacerbates issues with INH adduct formation. These findings provide detailed structural insights into how alterations at S315 impact both the heme‐binding site and substrate accessibility, offering a clearer understanding of the molecular basis of resistance.

### 3.2 Discrepancies in heme loading

Previous studies have shown that INH resistance mutations in KatG often impair heme loading and retention (Munir et al., 2021). Consistent with this, we observe reduced heme incorporation in all S315 variants analyzed in this study. Based on the number of particles in the final cryo‐EM maps, we were also able to work out the percentage of no, one, or two heme KatG maps (Table S6). However, we note discrepancies between UV–visible spectroscopy measurements and the apparent heme occupancy in the cryo‐EM reconstructions. This may be attributed to sample preparation artifacts during cryo‐EM, particularly the freezing process and exposure to the air‐water interface, which could dislodge loosely bound heme, although this has not been specifically referenced. Disruption of the H‐bond network may weaken heme binding, resulting in its partial or complete loss during vitrification. As such, while we cannot accurately quantify the exact heme content in each sample from the cryo‐EM data alone, we can conclude that heme binding in these variants is both lower and less stable compared to WT KatG and the S315T variant.

### 3.3 Cryo‐EM enables high‐resolution analysis of point mutations

A key advance of this study is the demonstration that cryo‐EM can be effectively used to resolve structural changes arising from single‐point mutations. It is often assumed that cryo‐EM lacks the resolution necessary to detect such subtle differences. However, with careful sample optimization and rigorous data processing, we show that high‐resolution cryo‐EM can indeed reveal precise structural alterations caused by point mutations. This capability opens new avenues for investigating structure–function relationships in proteins carrying clinically relevant mutations.

The current resolution record for cryo‐EM is approximately 1.22 Å for apoferritin, while most structures are typically resolved at 2.0–3.0 Å. At resolutions better than ~3 Å, side‐chain features become clearly discernible; however, for detailed analysis of single‐residue changes, resolutions below 2.5 Å are preferable. Achieving this requires optimal vitrification conditions and sufficiently large, high‐quality datasets.

While x‐ray crystallography can routinely achieve sub‐2 Å resolution, it often fails to capture the conformational heterogeneity and flexibility present within a sample. This represents one of the major strengths of cryo‐EM: its ability to visualize multiple conformational states and detect structural changes introduced by mutations that may shift or broaden the conformational landscape. Such effects can be missed by crystallography, which typically captures only the most thermodynamically stable state, one that may not accurately represent the full conformational ensemble of the protein.

### 3.4 Insights into the S315 INH binding site

The precise binding site of INH on KatG has been a longstanding topic of investigation. Our 2021 cryo‐EM study predicted that INH likely binds near residue S315, a hypothesis also supported by several other groups (Reyes et al., 2019; Srivastava et al., 2017). The current study, which characterizes various S315 mutations, strengthens this conclusion by demonstrating that even subtle alterations at this single residue can confer INH resistance. The detailed structural and functional insights provided here may guide the design of INH or INH derivatives tailored to overcome resistance in KatG variants.

### 3.5 Next‐generation anti‐TB therapeutics

Our findings provide a detailed molecular explanation for clinically observed isoniazid (INH) resistance in *M. tuberculosis* and establish a foundation for the rational design of next‐generation anti‐TB therapeutics. By elucidating how specific point mutations at Ser315 alter the conformation of the KatG enzyme, disrupting its catalytic efficiency and impairing INH activation, we highlight the critical structural determinants that underlie drug resistance. This mechanistic insight opens new avenues for targeted therapeutic development.

Future strategies could involve the design of INH analogues capable of overcoming steric hindrance imposed by Ser315 mutations, or the development of compounds engineered to interact more effectively with altered KatG access channels. Alternatively, small molecules that stabilize the active conformation of mutant KatG or exploit alternative activation pathways could represent promising directions. Collectively, these approaches may enable the creation of next‐generation therapeutics that retain efficacy against resistant *M. tuberculosis* strains and contribute to overcoming one of the major barriers in global TB control.

## 4 STAR MATERIALS AND METHODS

### 4.1 Over‐expression and purification of WT and S315 variants

KatG variants were either acquired from the University of Cambridge (WT and S315G) or were ordered through Genscript (S315T, S315I, S315N, S315R); each gene is located in a pHAT4 vector. KatG was expressed and purified based on protocols previously established by Munir et al. (2021). In brief, the protein was subjected to a three‐step purification protocol. Using the His‐tag located on the N‐terminus, the protein was bound to a HisTrap HP 5 mL (Cytiva). This was followed by anion affinity chromatography using a HiTrap Q column (Cytiva). The final step of the purification consists of size exclusion chromatography using a Superdex S200 10/300 Increase (Cytiva). Protein purity was checked throughout via a 10% SDS PAGE gel.

### 4.2 UV–visible spectroscopy of KatG and S315 variants

UV–visible spectrums of WT KatG and the S315 variants were measured using a Cary 300 spectrophotometer (Agilent), with a 1 cm path‐length quartz cuvette (Hellma) between wavelengths of 240–800 nm. The absorbance at 280 nm was measured, and concentrations were calculated using the Beer–Lambert law with an extinction coefficient (ε) of 163,290 M^−1^ cm^−1^.

### 4.3 Cryo‐EM grid preparation

Purified KatG variants were mixed with CHAPSO (3‐[(3‐Cholamidopropyl) dimethylammonio]‐1‐propanesulfonate) to a final concentration of 8 mM prior to 3 μL of the protein at ~3.5 mg/mL being applied to glow discharged (60 s at a current of 25 mA in a GloQube plus (Quorum)) on holey carbon grids (Quantifoil Cu R1.2/1.3, 300 mesh). The grids were then blotted with filter paper once to remove any excess sample and plunge‐frozen in liquid ethane using a FEI Vitrobot Mark IV (Thermo Fisher Scientific Ltd) at 4°C and 100% humidity.

### 4.4 Cryo‐EM data acquisition

All cryo‐EM data were collected at the Midlands Regional cryo‐EM facility, University of Leicester. The data were collected on a Titan Krios using the K3 detector (Gatan) and the EPU acquisition software (ThermoFisher Scientific). Data acquisition parameters are shown in Table S2.

### 4.5 Image processing

All data were processed in CryoSPARC (Punjani et al., 2017; Punjani & Fleet, 2021). In short, CTF correction, motion correction, and particle blob picking were first performed. These particles were extracted in a 288‐pixel box size, which were then subjected to two‐dimensional (2D) classification followed by ab initio reconstruction to generate initial 3D models. Each dataset contained some degree of heterogeneity and was processed differently. These are summarized within the cryo‐EM workflows given in Figures S5, S9–S12. Once maps were separated, those with high resolution were run through a reference‐based motion correction followed by all being run through a final non‐uniform refinement.

### 4.6 Structure refinement and model building

The final cryo‐EM maps were used for model building. The WT KatG cryo‐EM model (PDB: 7AG8) was used as an initial template and rigid‐body fitted into the cryo‐EM density in UCSF chimera (Pettersen et al., 2004). Namdinator (Kidmose et al., 2019) was used to adjust the structure, and several rounds of real‐space refinement were then performed in Phenix (Afonine et al., 2018). The heme ligand was read in via Coot (Emsley et al., 2010), and regions adjusted according to Phenix. All structures were refined and validated before being deposited into the PDB and EMDB with accession codes given in Table S2.

### 4.7 Caver analysis

Caver 3.0 was used to measure the size of the substrate access channel. Each KatG variant used the same parameters to generate tunnels. In order to specify the exact location for tunnel generation, the following residues were specified: X315, P232, D137, R104, W107, H108, and Heme. The parameters used to generate the tunnels are shown in Table S5.

## AUTHOR CONTRIBUTIONS

**Thomas Allport:** Investigation; methodology; validation; visualization; software; formal analysis; data curation. **Amanda K. Chaplin:** Conceptualization; writing – original draft; writing – review and editing; supervision; resources; project administration; funding acquisition.

## FUNDING INFORMATION

We thank the F100 University of Leicester PhD studentship for support of T.A.

## CONFLICT OF INTEREST STATEMENT

The authors declare no competing interests.

## Supporting information


**Supplementary Figure 1:** Catalase and peroxidase reaction scheme and activation of isoniazid by KatG. (a) Simplified reaction scheme of KatG catalase (blue) and peroxidase (purple), both reactions are initiated through the formation of compound I (red). Compound I is reduced back to resting state Fe^3+^ heme through either a two‐electron transfer or a two‐step electron transfer depending on if it is the catalase or peroxidase pathway. (b) Key steps in the activation of isoniazid (INH) and the formation of the INH‐NAD adduct.
**Supplementary Figure 2:** SDS PAGE gel of KatG WT and S315 variants. 10% SDS PAGE gel of KatG S315 variants and WT with each producing a dominant band at ~80 kDa corresponding to a KatG monomer.
**Supplementary Table 1:** Soret and Q bands (wavelength, nm) for WT and S315 KatG variants.
**Supplementary Figure 2:** Cryo‐EM maps and models for the five S315 mutants. Protomer A is in light blue, protomer B in dark blue. Where heme is present it is highlighted.
**Supplementary Table 2:** Cryo‐EM data collection and refinement parameters.
**Supplementary Figure 4:** Patch motion corrected and 2D classes of S315 variants. (a) S315T corrected micrograph and example 2D classes. (b) S315R corrected micrograph and example 2D classes. (c) S315I corrected micrograph and example 2D classes. (d) S315N corrected micrograph and example 2D classes. (e) S315G corrected micrograph and example 2D classes.
**Supplementary Figure 5:** CryoSPARC processing pipeline KatG S315T. Following the initial pre‐processing jobs both good and junk 2D classes were generated these produced 3 initial 3D reconstructions which were combined in a heterogenous refinement. The map resembling KatG was further processed via another heterogenous refinement separating out two heme and one heme bound structures. These two maps were processed separately through a homogenous and non‐uniform refinement due to the high resolution S315T two heme was refence based motion corrected producing a 2.27 Å map
**Supplementary Figure 6:** Local resolution estimation and global FSC curves KatG variants, (a) local resolution and FSC curve of S315T, one heme. (b) Local resolution estimation and FSC curves of S315R, two heme and one heme. (c) local resolution and FSC curve of S315G, one heme and no heme. (d) local resolution and FSC curve of S315N, one heme and no heme. (e) Local resolution and FSC curve of S315I, two heme, one heme and no heme.
**Supplementary Figure 7:** Superposition of KatG S315 variants against WT. Comparison of WT 7AG8 (gray) against each KatG variant, S315R (purple), S315T (turquoise), S315N (green), S315G (red), S315I (orange).
**Supplementary Figure 8:** Superposition of KatG S315 variants against each other. Comparison of each KatG variants active site against each other, S315R (purple), S315T (turquoise), S315N (green), S315G (red), S315I (orange).
**Supplementary Table 3:** Table showing the catalase activity of WT and S315 variants produced from Bagcchi (2023).
**Supplementary Table 4:** Table showing the Peroxidase activity of WT and S315 variants produced from Bagcchi (2023).
**Supplementary Figure 9:** CryoSPARC processing pipeline KatG S315G. Following the initial pre‐processing jobs both good and junk 2D classes were generated due to the high number of particles only the good 2D classes were carried out for an initial 3D reconstruction producing 3 maps the map resembling KatG was taken further via a homogenous refinement, initial heterogenous refinements were unsuccessful due to the hook region being the target of separation. To alleviate this a mask of the hook not present was made this was used to locally refine out the hook. Following this the no heme map was inputted into a 3D classification which separated out a no heme and one heme bound state. These were processed separately through a non‐uniform refinement and reference‐based motion correction.
**Supplementary Figure 10:** CryoSPARC processing pipeline KatG S315I. Following the initial pre‐processing jobs both good and junk 2D classes were generated these produced 2 initial 3D reconstruction for the good classes and 3 for junk 2D classes these were combined for a heterogenous refinement. The map resembling KatG was taken for further processing due to the high heterogeneity both 3D variability and 3D classification was employed to separate no heme and two heme maps from the one heme map.
**Supplementary Figure 11:** CryoSPARC processing pipeline KatG S315N. Following the initial pre‐processing jobs both good and junk 2D classes were generated these produced 2 initial 3D reconstruction for the good classes and 3 for junk 2D classes these were combined for a heterogenous refinement. The map resembling KatG was taken for further processing and was ran through a heterogenous refinement separating the one heme and no heme map. These two maps were then processed separately through non‐uniform refinement and reference‐based motion correction to similar resolution.
**Supplementary Figure 12:** CryoSPARC processing pipeline KatG S315R. Following the initial pre‐processing jobs both good and junk 2D classes were generated due to the high number of particles only the good 2D classes were carried out for an initial 3D reconstruction producing 3 maps the map resembling KatG was taken further via heterogenous refinement separating out two maps. The map of better resolution was inputted into a 3D classification of 2 classes separating out a one heme and two heme map. These two maps were processed separately through a non‐uniform refinement and reference‐based motion correction producing two maps of similar resolution.
**Supplementary Table 5:** Caver 3.0 analysis parameters (Jagielski et al., 2014).
**Supplementary Table 6:** Cryo‐EM particle analysis.

## Data Availability

The data that support the findings of this study are openly available in PDB at https://www.google.co.uk/search?q=PDB&sca_esv=de6549fe37924183&source=hp&ei=Zh4YadHbFeyrhbIP0ryawQo&iflsig=AOw8s4IAAAAAaRgsdkke3rdh_579‐QuUqCBRr7Zv3wR6&ved=0ahUKEwjRpvWuxPOQAxXsVUEAHVKeJqgQ4dUDCBo&uact=5&oq=PDB&gs_lp=Egdnd3Mtd2l6IgNQREIyCxAAGIAEGLEDGIMBMg. All structural data presented are publicly available. Cryo‐EM structures and maps are deposited at the PDB and EMDB with accession codes as follows: S315T 9SGL, EMD‐54872; S316I (Two heme), 9SGM, EMD‐54873; S315I (No) 9SGN, EMD54874; S315I (One) 9SGO, EMD‐54875; S315R (Two) 9SGP, EMD‐54876; S315R (One) 9SGQ, EMD‐54877; S315N (No) 9SGR, EMD‐54878; S315N (One) 9SGY, EMD‐54882; S315G (No) 9SGS, EMD‐54879; S315G (One) 9SGT, EMD‐54880.
