# Supplementary material for: Uncovering the structural impact of KatG Ser315 mutations in Mycobacterium tuberculosis via cryo‐EM
Source: Protein Sci. 2025 Dec 23;35(1):e70409. doi: 10.1002/pro.70409 (PMC12724002; doi:10.1002/pro.70409)
Supplement: Supplementary file 1 — Supplementary Figure 1: Catalase and peroxidase reaction scheme and activation of isoniazid by KatG. (a) Simplified reaction scheme of KatG catalase (blue) and peroxidase (purple), both reactions are initiated through the formation of compound I (red). Compound I is reduced back to resting state Fe3+ heme through either a two‐electron transfer or a two‐step electron transfer depending on if it is the catalase or peroxidase pathway. (b) Key steps in the activation of isoniazid (INH) and the formation of the INH‐NAD adduct. Supplementary Figure 2: SDS PAGE gel of KatG WT and S315 variants. 10% SDS PAGE gel of KatG S315 variants and WT with each producing a dominant band at ~80 kDa corresponding to a KatG monomer. Supplementary Table 1: Soret and Q bands (wavelength, nm) for WT and S315 KatG variants. Supplementary Figure 2: Cryo‐EM maps and models for the five S315 mutants. Protomer A is in light blue, protomer B in dark blue. Where heme is present it is highlighted. Supplementary Table 2: Cryo‐EM data collection and refinement parameters. Supplementary Figure 4: Patch motion corrected and 2D classes of S315 variants. (a) S315T corrected micrograph and example 2D classes. (b) S315R corrected micrograph and example 2D classes. (c) S315I corrected micrograph and example 2D classes. (d) S315N corrected micrograph and example 2D classes. (e) S315G corrected micrograph and example 2D classes. Supplementary Figure 5: CryoSPARC processing pipeline KatG S315T. Following the initial pre‐processing jobs both good and junk 2D classes were generated these produced 3 initial 3D reconstructions which were combined in a heterogenous refinement. The map resembling KatG was further processed via another heterogenous refinement separating out two heme and one heme bound structures. These two maps were processed separately through a homogenous and non‐uniform refinement due to the high resolution S315T two heme was refence based motion corrected producing a 2.27 Å map Supplementary Figu [file PRO-35-e70409-s001.pdf]

Supplementary figures

Uncovering the Structural Impact of KatG Ser315 Mutations in *Mycobacterium tuberculosis* via Cryo-EM

Thomas Allport[1] and Amanda K. Chaplin[1]*.

1. Leicester Institute for Structural and Chemical Biology, Department of Molecular and Cell Biology, University of Leicester; Leicester, UK.

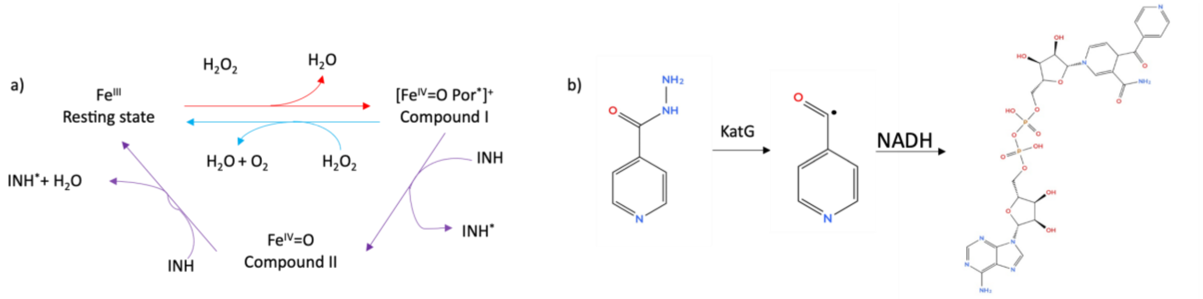

Supplementary Figure 1: Catalase and peroxidase reaction scheme and activation of isoniazid by KatG. a) Simplified reaction scheme of KatG catalase (blue) and peroxidase (purple), both reactions are initiated through the formation of compound I (red). Compound I is reduced back to resting state Fe^{3+} heme through either a two-electron transfer or a two-step electron transfer depending on if it is the catalase or peroxidase pathway. **b)** key steps in the activation of isoniazid (INH) and the formation of the INH-NAD adduct.

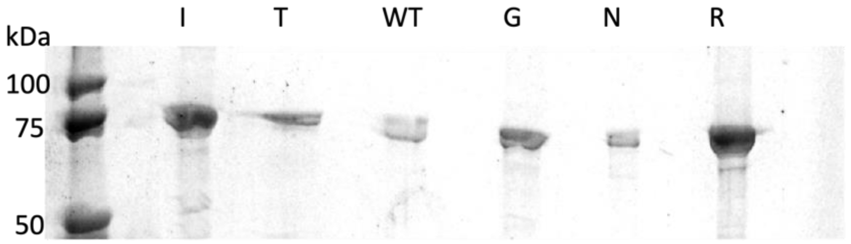

Supplementary Figure 2: SDS PAGE gel of KatG WT and S315 variants. 10% SDS PAGE gel of KatG S315 variants and WT with each producing a dominant band at ~80 kDa corresponding to a KatG monomer.

Supplementary Table 1: Soret and Q bands (wavelength, nm) for WT and S315 KatG variants.

	Soret (nm)	Q bands (nm)	
		α	β
WT	408	636	519
S315G	409	634	531
S315I	407	637	505
S315N	409	632	516
S315R	407	638	507
S315T	406	634	500

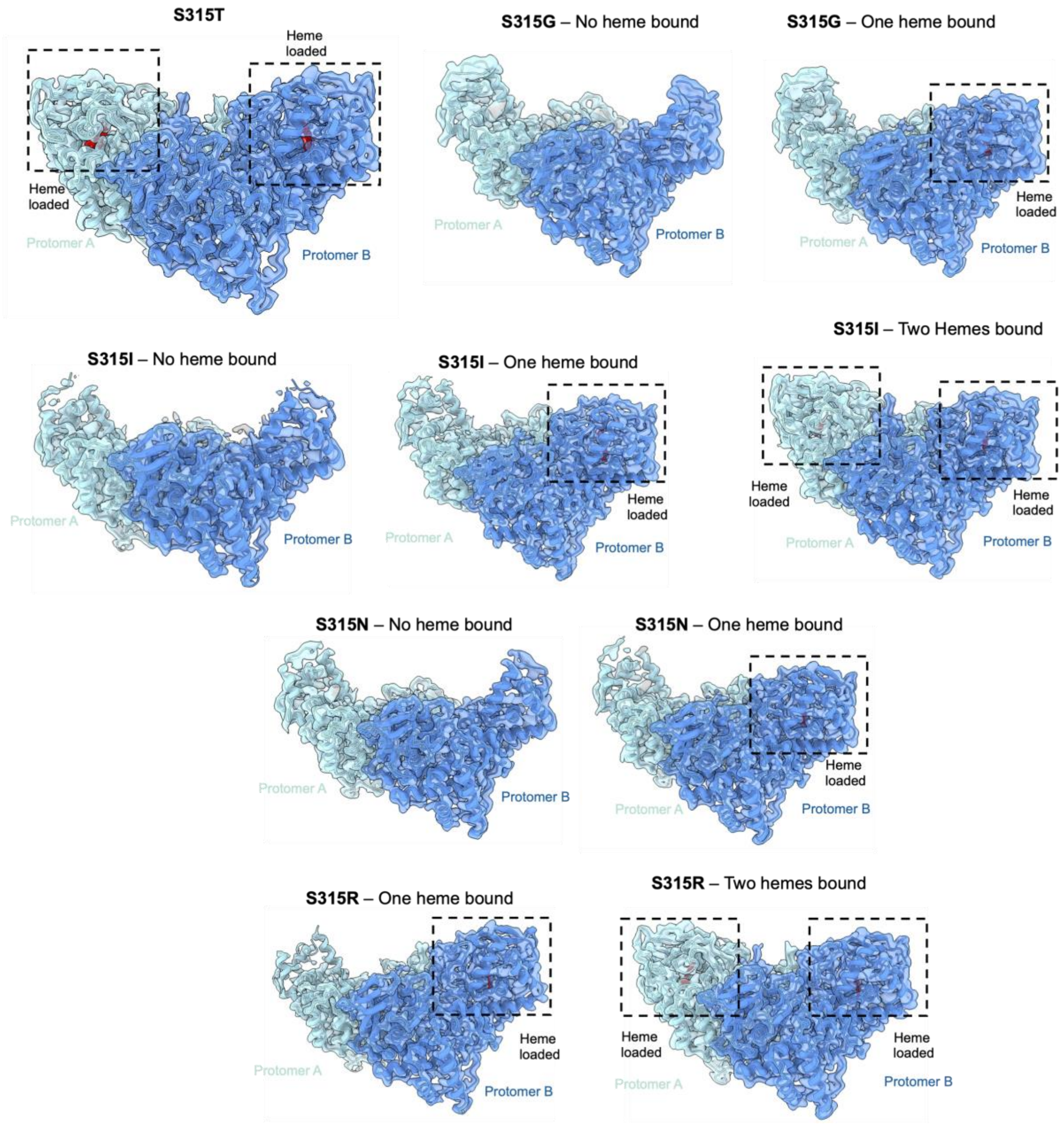

Supplementary Figure 3: Cryo-EM maps and models for the five S315 mutants.
Protomer A is in light blue, protomer B in dark blue. Where heme is present it is highlighted.

Supplementary Table 2: Cryo-EM data collection and refinement parameters.

Data collection	S315G		S315I			S315N		S315R		S315T
Magnification	105k		105k			105k		105k		105k
Flux on detector	17		14.9			14.9		15.7		16.8
Energy Filter Slit width	20		20			20		20		20
Accelerating voltage	300		300			300		300		300
Spherical aberration	2.7		2.7			2.7		2.7		2.7
Number of fractions	50		50			50		50		50
Exposure time	2		2.2			2.2		2.2		2
Total exposure dose	60		48.279			48.279		50.871		49.486
Dose per frame	1.2		0.966			0.966		1.018		0.9897
Targeted defocus range	-2.5 to -1.0		-2.2 to -0.8			-2.2 to -0.8		-2.2 to -0.8		-2.2 to -0.8
Calibrated pixel size	0.824		0.824			0.824		0.824		0.824
**Data processing**	**no heme**	**one heme**	**no heme**	**one heme**	**two heme**	**no heme**	**one heme**	**one heme**	**two heme**	**two heme**
**extraction box size**	288	288	288	288	288	288	288	288	288	288
**initial particles**	759,062	759,062	491,911	491,911	491,911	298,153	298,153	120,621	120,621	379,047
**final particles**	290,374	266,376	38,335	201,107	30,883	79,721	96,771	41,108	41,894	227,266
**Symmetry**	C2	C1	C1	C1	C1	C2	C1	C1	C1	C1
**Refinement**	**no heme PDB: EMDB:**	**one heme PDB: EMDB:**	**no heme PDB: EMDB:**	**one heme PDB: EMDB:**	**two heme PDB: EMDB:**	**no heme PDB: EMDB:**	**one heme PDB: EMDB:**	**one heme PDB: EMDB:**	**two heme PDB: EMDB:**	**two heme PDB: EMDB:**
**Map resolution at FSC = 0.143**	2.7	2.7	3.0	2.3	2.8	2.6	2.6	2.7	2.6	2.2
**Model composition**										
**Non-hydrogen atoms**	8646	9837	8270	9676	10989	8303	9528	9611	11034	11019
**Protein residues**	1134	1284	1098	1265	1434	1099	1259	1258	1434	1434
**B factor (Å^2)**										
**Protein**	150.90	152.16	109.73	107.67	128.35	131.44	127.26	121.40	121.90	99.55
**Ligand (heme)**	N/A	161.62	N/A	99.63	126.58	N/A	125.16	102.36	118.31	87.07
**RMSD**										
**Bond lengths (Å)**	0.002	0.002	0.003	0.002	0.004	0.003	0.003	0.002	0.004	0.003
**Bond angles (°)**	0.528	0.479	0.516	0.460	0.560	0.573	0.489	0.529	0.497	0.516
**Validation**										
**MolProbity score**	1.47	1.44	1.53	1.38	1.66	1.89	1.32	1.49	1.50	1.51
**Clashscore**	4.67	5.71	6.32	5.54	6.86	5.73	5.60	6.16	6.49	5.01
**Poor rotamers (%)**	0.69	1.22	0.73	0.62	0.73	2.43	0.64	0.73	0.46	0.46
**Ramachandran plot (%)**										
**Favored**	96.49	97.72	96.94	97.61	95.87	95.83	97.91	97.19	97.20	96.29
**Allowed**	3.42	2.05	2.88	2.39	3.85	3.99	2.09	2.65	2.73	3.64
**Disallowed**	0.09	0.24	0.19	0.00	0.28	0.19	0.00	0.16	0.07	0.07

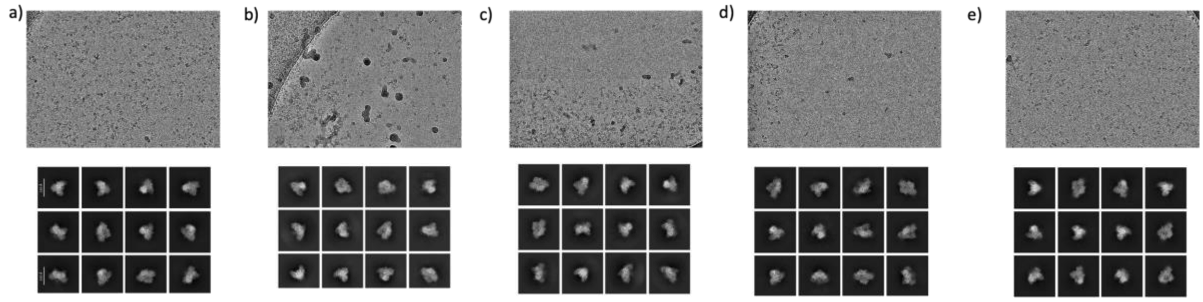

Supplementary Figure 4: Patch motion corrected and 2D classes of S315 variants. a) S315T corrected micrograph and example 2D classes. **b)** S315R corrected micrograph and example 2D classes. **c)** S315I corrected micrograph and example 2D classes. **d)** S315N corrected micrograph and example 2D classes. **e)** S315G corrected micrograph and example 2D classes.

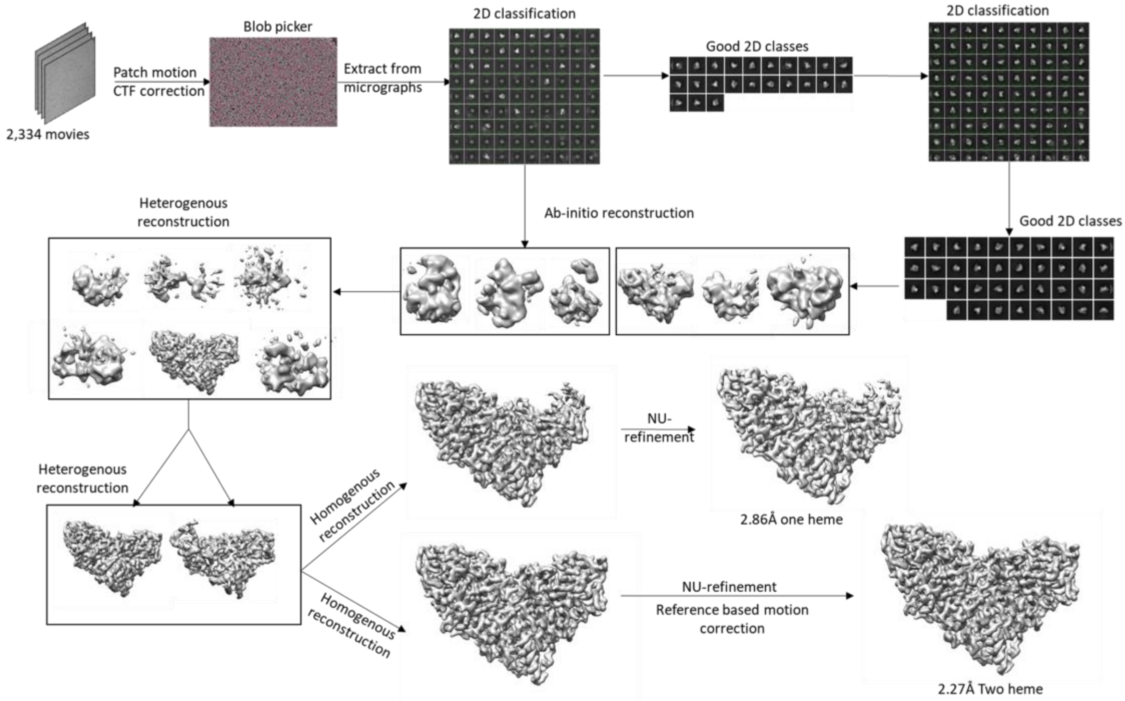

Supplementary Figure 5: CryoSPARC processing pipeline KatG S315T. Following the initial pre-processing jobs both good and junk 2D classes were generated these produced 3 initial 3D reconstructions which were combined in a heterogenous refinement. The map resembling KatG was further processed via another heterogenous refinement separating out two heme and one heme bound structures. These two maps were processed separately through

a homogenous and non-uniform refinement due to the high resolution S315T two heme was refence based motion corrected producing a 2.27 Å map

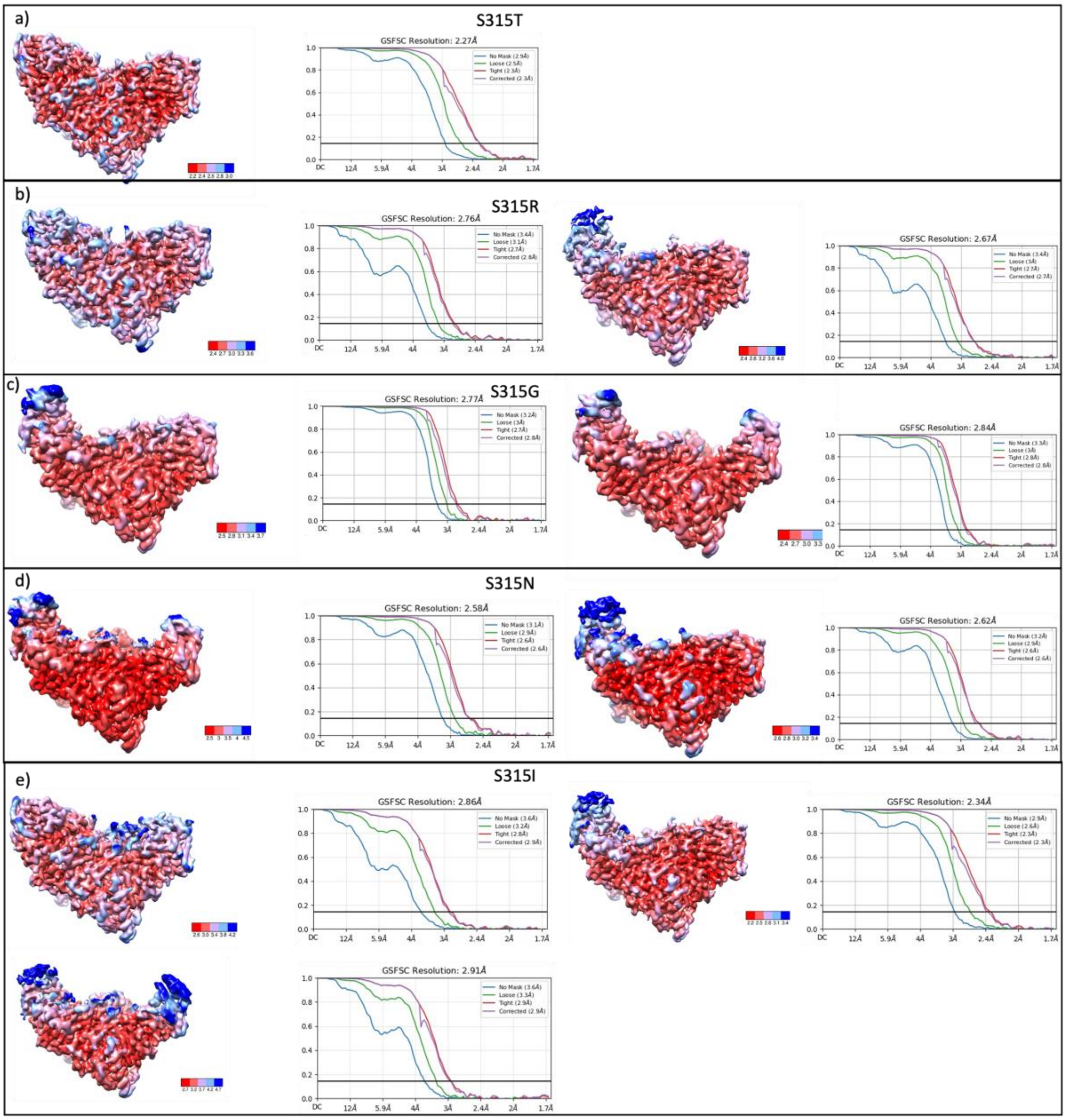

Supplementary Figure 6: Local resolution estimation and global FSC curves KatG variants, a) local resolution and FSC curve of S315T, one heme. **b)** Local resolution estimation and FSC curves of S315R, two heme and one heme. **c)** local resolution and FSC curve of S315G, one heme and no heme. **d)** local resolution and FSC curve of S315N, one heme and no heme. **e)** Local resolution and FSC curve of S315I, two heme, one heme and no heme.

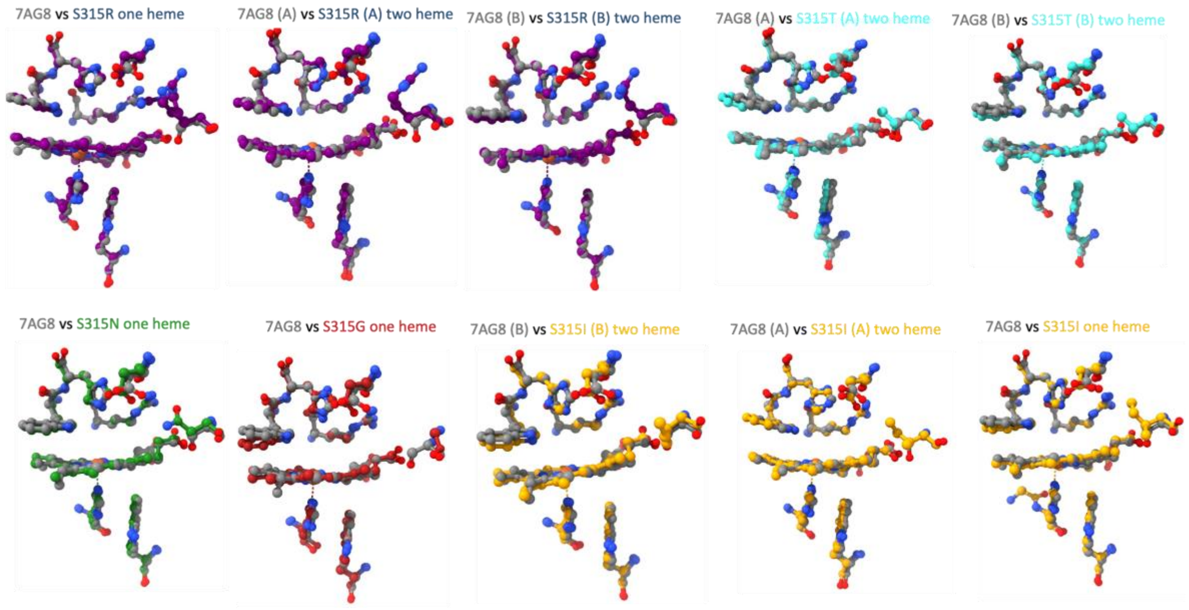

Supplementary Figure 7: Superposition of KatG S315 variants against WT. Comparison of WT 7AG8 (grey) against each KatG variant, S315R (purple), S315T (turquoise), S315N (green), S315G (red), S315I (orange).

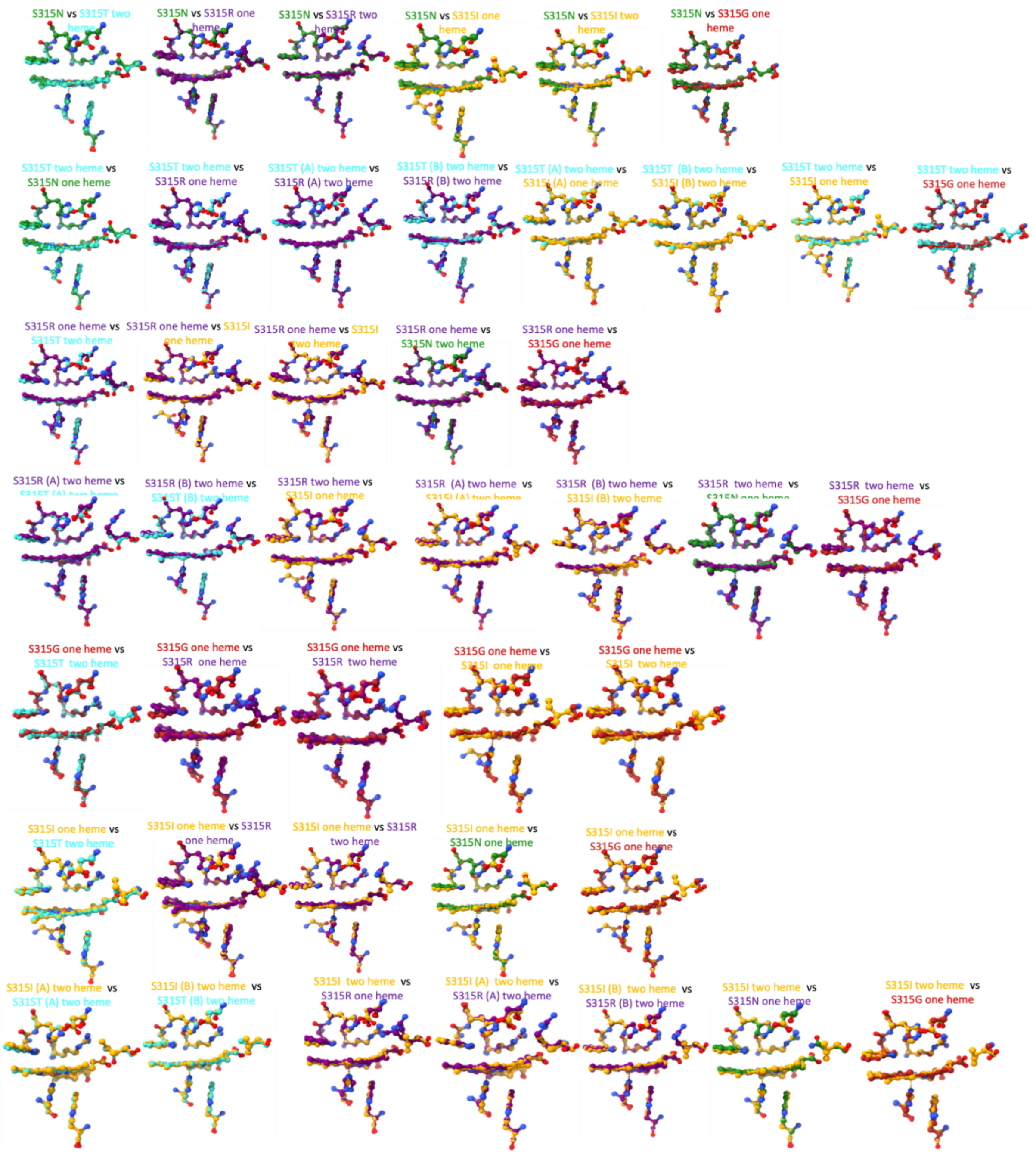

Supplementary Figure 8: Superposition of KatG S315 variants against each other. Comparison of each KatG variants active site against each other, S315R (purple), S315T (turquoise), S315N (green), S315G (red), S315I (orange).

Supplementary Table 3: Table showing the Catalase activity of WT and S315 variants produced from [1].

Catalase			
Mutant	k_{cat} (s^{-1})	K_m (mM)	k_{cat}/K_m (M^{-1} s^{-1})
WT	6000 ± 70	2.5 ± 0.2	(2.4 ± 0.028) × 10^6
S315G	711 ± 22	1.7 ± 0.3	(4.18 ± 0.76) × 10^5
S315I	739.3 ± 4.9	2.2 ± 0.06	(3.36 ± 0.10) × 10^5
S315N	326.5 ± 4.0	2.1 ± 0.2	(1.55 ± 0.15) × 10^5
S315R	727 ± 77	5.7 ± 2.1	(1.28 ± 0.49) × 10^5
S315T	2106 ± 47	3.9 ± 0.3	(5.40 ± 0.43) × 10^5

Supplementary Table 4: Table showing the Peroxidase activity of WT and S315 variants produced from [1].

Peroxidase			
Mutant	k_{cat} (s^{-1})	K_m (mM)	k_{cat}/K_m (M^{-1} s^{-1})
WT	0.062 ± 0.001	8.44 ± 0.45	7.3 ± 0.4
S315G	0.81 ± 0.02	26.5 ± 2.8	30.5 ± 3.3
S315I	0.61 ± 0.03	12.6 ± 2.6	48.1 ± 10.2
S315N	0.41 ± 0.02	20.7 ± 2.9	20.0 ± 2.9
S315R	0.64 ± 0.02	12.4 ± 2.4	51.9 ± 10.2
S315T	0.051 ± 0.003	2.06 ± 0.64	5.7 ± 1

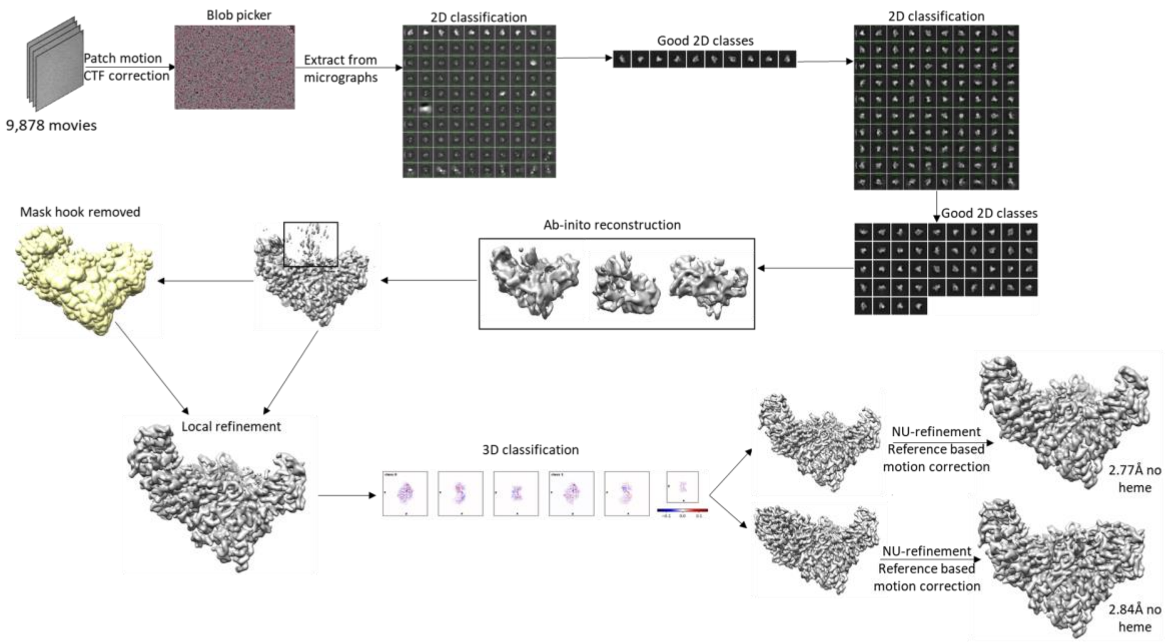

Supplementary Figure 9: CryoSPARC processing pipeline KatG S315G. Following the initial pre-processing jobs both good and junk 2D classes were generated due to the high number of particles only the good 2D classes were carried out for an initial 3D reconstruction producing 3 maps the map resembling KatG was taken further via a homogenous refinement, initial heterogenous refinements were unsuccessful due to the hook region being the target of separation . To alleviate this a mask of the hook not present was made this was used to locally refine out the hook. Following this the no heme map was inputted into a 3D classification which separated out a no heme and one heme bound state. These were processed separately through a non-uniform refinement and reference-based motion correction.

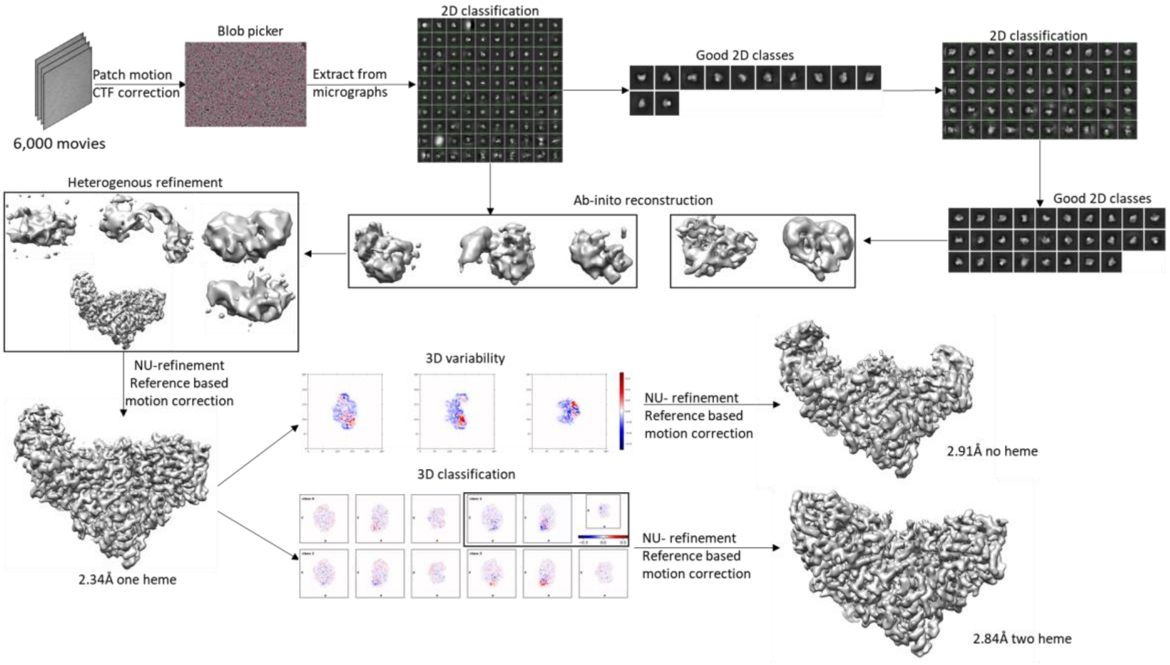

Supplementary Figure 10: CryoSPARC processing pipeline KatG S315I. Following the initial pre-processing jobs both good and junk 2D classes were generated these produced 2 initial 3D reconstruction for the good classes and 3 for junk 2D classes these were combined for a heterogenous refinement. The map resembling KatG was taken for further processing due to the high heterogeneity both 3D variability and 3D classification was employed to separate no heme and two heme maps from the one heme map.

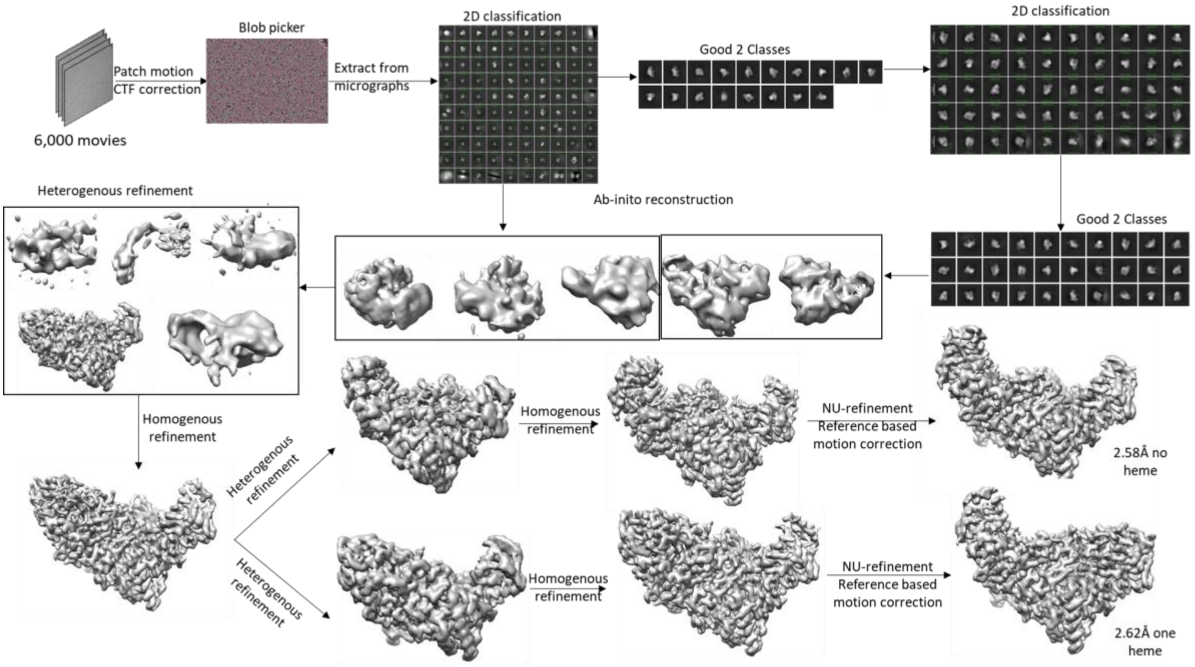

Supplementary Figure 11: CryoSPARC processing pipeline KatG S315N. Following the initial pre-processing jobs both good and junk 2D classes were generated these produced 2 initial 3D reconstruction for the good classes and 3 for junk 2D classes these were combined for a heterogenous refinement. The map resembling KatG was taken for further processing and was ran through a heterogenous refinement separating the one heme and no heme map. These two maps were then processed separately through non-uniform refinement and reference-based motion correction to similar resolution.

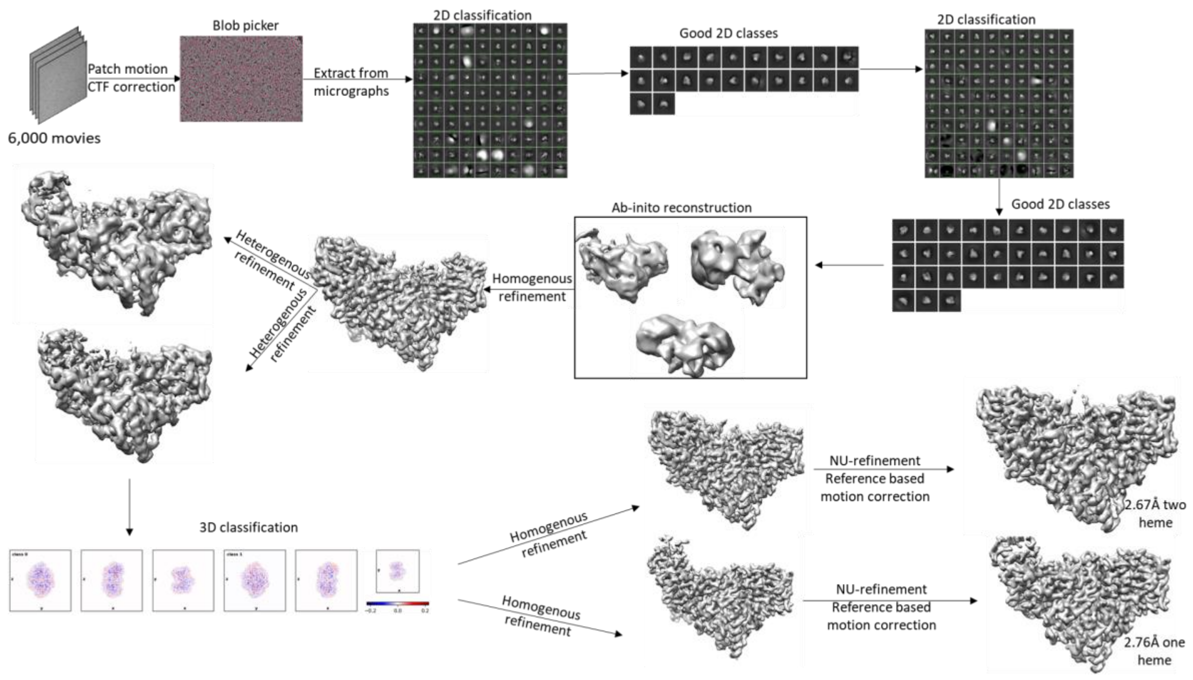

Supplementary Figure 12: CryoSPARC processing pipeline KatG S315R. Following the initial pre-processing jobs both good and junk 2D classes were generated due to the high number of particles only the good 2D classes were carried out for an initial 3D reconstruction producing 3 maps the map resembling KatG was taken further via heterogenous refinement separating out two maps. The map of better resolution was inputted into a 3D classification of 2 classes separating out a one heme and two heme map. These two maps were processed separately through a non-uniform refinement and reference-based motion correction producing two maps of similar resolution.

Supplementary Table 5: Caver 3.0 analysis parameters [2].

Caver Analysis Parameters	
Probe radius	0.4
Shell depth	6
Shell radius	4
Clustering threshold	5
Maximum distance (Å)	4
Desired radius (Å)	5

Supplementary Table 6: Cryo-EM particle analysis

Mutant	S315G			S315I				S315N			S315R			S315T	
Number of hemes	No	One	Total	No	One	Two	Total	No	One	Total	One	Two	Total	Two	Total
Particles	290,374	266,376	556,750	38,335	201,107	30,883	270,325	79,721	96,771	176,492	41,108	41,894	83,002	227,266	227,266
Ratio	0.52155	0.47845		0.14181	0.74395	0.11424		0.4517	0.5483		0.49527	0.50473		1	
Percentage	52.1552	47.8448		14.1811	74.3945	11.4244		45.1698	54.8302		49.5265	50.4735		100	

References

1 Cade, C. E., Dlouhy, A. C., Medzihradszky, K. F., Salas-Castillo, S. P. & Ghiladi, R. A. *Protein Sci* **19**, 458-474, (2010).

2 Chovancova, E., Pavelka, A., Benes, P., Strnad, O., Brezovsky, J., Kozlikova, B., Gora, A., Sustr, V., Klvana, M., Medek, P. *et al. PLoS Comput Biol* **8**, e1002708, (2012).
